# Diabetic kidney disease: the kidney disease relevant to individuals with diabetes

**DOI:** 10.1007/s10157-024-02537-z

**Published:** 2024-07-20

**Authors:** Keizo Kanasaki, Kohjiro Ueki, Masaomi Nangaku

**Affiliations:** 1 Department of Internal Medicine 1, Faculty of Medicine, Shimane University, 89-1 Enya-Cho, Izumo, 693-8501 Japan; 2 The Center for Integrated Kidney Research and Advance, Faculty of Medicine, Shimane University, 89-1 Enya-Cho, Izumo, 693-8501 Japan; 3 Diabetes Research Center, Research Institute, National Center for Global Health and Medicine, Tokyo, Japan; 4 Division of Nephrology and Endocrinology, The University of Tokyo Graduate School of Medicine, Tokyo, Japan

**Keywords:** Diabetic kidney disease, Diabetic nephropathy, Urine albumin, eGFR

## Abstract

In individuals with diabetes, chronic kidney disease (CKD) is a major comorbidity. However, it appears that there is worldwide confusion regarding which term should be used to describe CKD complicated with diabetes: diabetic nephropathy, diabetic kidney disease (DKD), CKD with diabetes, diabetes and CKD, etc. Similar confusion has also been reported in Japan. Therefore, to provide clarification, the Japanese Diabetes Society and the Japanese Society of Nephrology collaborated to update the corresponding Japanese term to describe DKD and clearly define the concept of DKD. In this review, we briefly described the history of kidney complications in individuals with diabetes and the Japanese definition of the DKD concept and provided our rationale for these changes.

## Kidney diseases in individuals with diabetes: a historical perspective

Our predecessors have utilized urine as a biomarker to understand the pathophysiology of diverse diseases. In the context of diabetes mellitus, the Italian physician Domenico Cotugno suggested the formation of coagulants upon heating urine and reported the presence of proteinuria-like materials [1]. Approximately two centuries later, Viberti et al. [2] and Mogensen et al. [3] reported the significance of albuminuria as a biomarker for diabetic nephropathy in individuals with type 1 and type 2 diabetes. Kimmelstiel and Wilson first described nodular lesions in diabetic nephropathy in 1936 [4]. As early as 1916, Nagayo, a pathologist at the University of Tokyo, confirmed pathological changes in the kidney among individuals with diabetes by dissecting the body of the renowned Japanese author Soseki Natsume. In 1927, Nagayo made a seminal report of characteristic kidney changes in three autopsies of cases with diabetes [5].

**Fig. 1:**
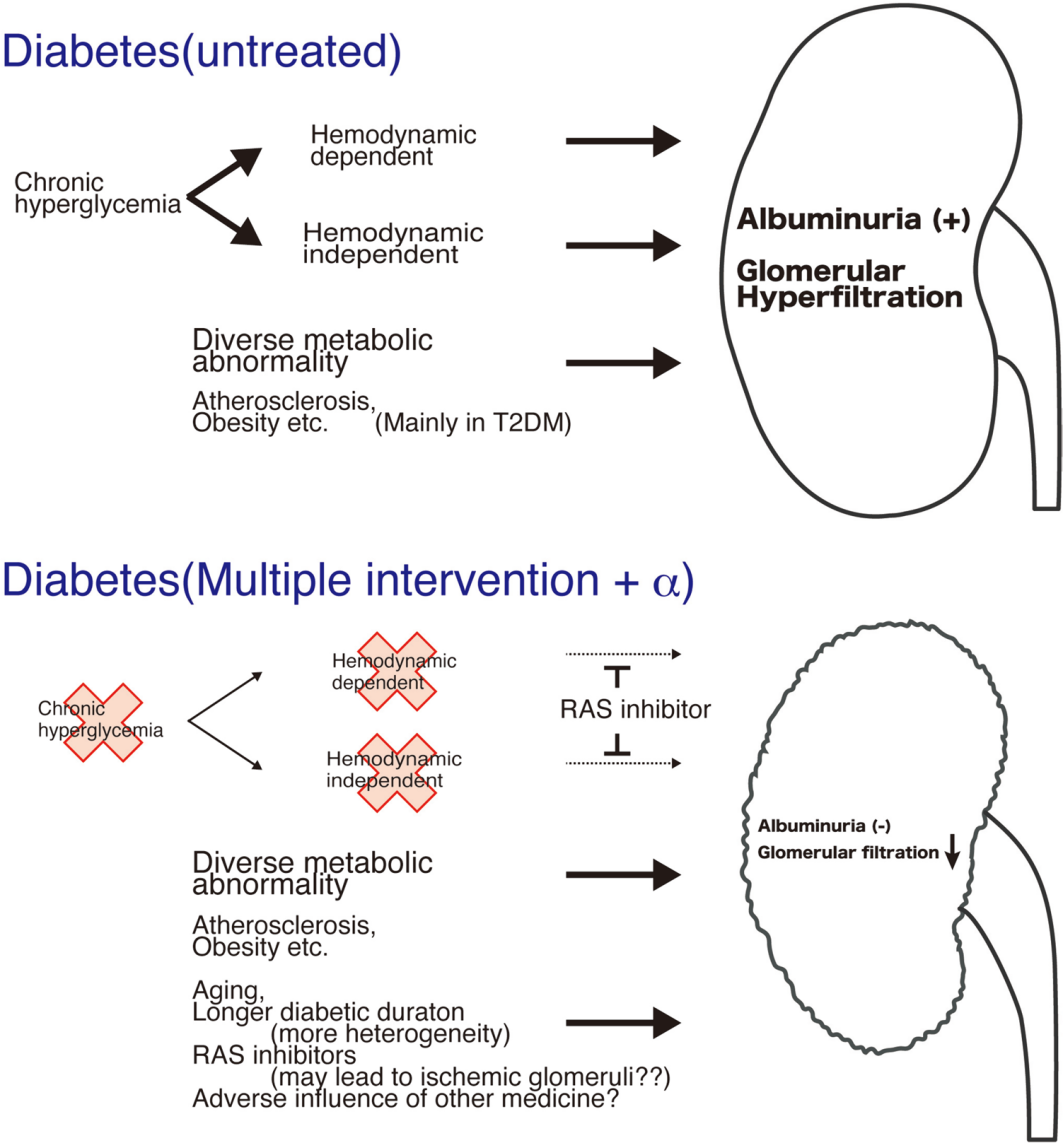
Kidney phenotypes in diabetes: untreated and post-multifactorial intervention (modified from reference [31]). In the untreated diabetic state (upper), chronic hyperglycemia is correlated with both pathological and functional kidney abnormalities. These arise via mechanisms that are both hemodynamically dependent and independent. Conversely, in the scenario of multifactorial interventions (lower), although traditional kidney damages have been mitigated, other health problems have emerged

In 1921, insulin was discovered in pancreatic extract by Banting and Best, and the clinical application of insulin was initiated in 1922. Therefore, the kidney alterations in these cases with diabetes reported by Nagayo [5] and Kimmelstiel and Wilson [4] were likely manifestations of untreated diabetes, without adequate therapeutic interventions to prevent the progression of kidney disease. Furthermore, when Viberti [2] and Mogensen [3] reported the significance of albuminuria as a biomarker for diabetic nephropathy, the treatment approaches fundamentally differed from those used in the current era, notably before the era of renin-angiotensin system (RAS) inhibitors. Over the extensive analysis of “urine” and “kidney pathological findings,” clinical practice and research on diabetic nephropathy have been established; however, most of these advances have been built on the foundation of observations of type 1 diabetes with insufficient blood glucose managements and untreated type 2 diabetes (Fig. 1).

## Alterations in kidney complications in the individuals with diabetes

In the typical course of diabetic nephropathy, an increase in urinary albumin excretion is observed after a certain duration of diabetes, followed by intermittent to sustained proteinuria and a gradual decline in kidney function, leading to chronic kidney failure and ultimately end-stage kidney disease. Early detection and staging of diabetic nephropathy are impossible without urinary albumin levels. Additionally, albuminuria positive individuals with diabetes are at high risk for kidney and/or cardiovascular outcomes. A research team at Kanazawa University indicated that biopsy-proven diabetic nephropathy cases with increased urinary albumin levels displayed significant deterioration in kidney function [6]. In the EMPA-Kidney trial (The Study of Heart and Kidney Protection With Empagliflozin [ClinicalTrials.gov ID NCT03594110]), placebo-treated cases in the normal urinary albumin group (urinary albumin-to-creatinine ratio [ACR] < 30 mg/g Cre) exhibited a significantly slower rate of decline in kidney function than did those in the positive group (ΔeGFR: macroalbuminuria ([ACR] ≥ 300 mg/g Cre) − 4.11 ml/min/1.73 m^2^/year, microalbuminuria group (300 > [ACR] ≥ 30 mg/g Cre) − 1.69 ml/min/1.73 m^2^/year, normal urinary albumin group − 0.89 ml/min/1.73 m^2^/year) [7]. The presence of albuminuria was attributed to the higher kidney and cardiovascular risk in individuals with diabetes in the JDDM54 study [8]. Cohort data of Japanese individuals with diabetes clearly showed that the higher albuminuria is at a high risk for both kidney and cardiovascular outcomes [9]. Unfortunately, in Japan, urinary albumin levels are routinely monitored in only approximately 20% of individuals with diabetes. Here, the authors would like to emphasize the need for awareness of the clinical significance of urinary albumin measurements [10].

However, it is becoming clear that the clinical course of kidney complications in individuals with diabetes does not necessarily follow the typical course of diabetic nephropathy. Afkarian and colleagues conducted a comprehensive investigation on kidney complications in individuals with diabetes aged ≥ 20 years in the United States from 1988 to 2014, including analysis of treatment drugs (National Health and Nutrition Examination Surveys: NHANES) [11]. They found significant reductions in HbA1c levels, systolic blood pressure, LDL cholesterol, and triglycerides, as well as an increase in the proportion of cases prescribed blood glucose-lowering drugs, RAS inhibitors, and statins across the survey periods (1988–1994, 1999–2004, 2005–2008, 2009–2014). The authors defined diabetic kidney disease (DKD_NHANES_) as albuminuria equal to or greater than microalbuminuria or an eGFR < 60 ml/min/1.73 m^2^, and they reported no significant change in the proportion of DKD_NHANES_ cases from 1988–1994 to 2009–2014. However, there was a decreasing trend in albuminuria-positive cases (ACR ≥ 30 mg/g Cre) and cases with macroalbuminuria (ACR ≥ 300 mg/g Cre), while the number of cases with an eGFR < 60 ml/min/1.73 m^2^ significantly increased during the same period. The authors suggested that improved glycemic control and the use of RAS inhibitors were potential reasons for the decrease in albuminuria from 1999 to 2014. They also speculated that the lack of a reduction in albuminuria in either African American or Mexican American individuals might be due to less opportunity for evidence-based treatment practices in these populations (high blood glucose, low prescription of RAS inhibitors). Although there is insufficient evidence to explain the secular trend of decreased eGFR, the authors considered mechanisms associated with alterations in kidney hemodynamics, such as suppression of intraglomerular pressure, due to RAS inhibitors or decreased systemic blood pressure. Furthermore, the authors acknowledged that a prolonged duration of diabetes could be associated with kidney damage [11]. Similar secular changes in the treatment options and in the kidney damage phenotype have been shown by Shiga University of Medical Science [12] and Tokyo Women’s Medical University [13]. Recent cluster analyses in individuals with diabetes have demonstrated that clusters with high insulin resistance, obesity, and accumulation of visceral fat are more prone to the risk for future kidney damage than clusters with poor blood glucose control and β-cell dysfunction [14–16]. The alteration in the landscape of kidney complications in individuals with diabetes over time is likely influenced by advances in treatments aimed at controlling complications. Furthermore, various comorbidities associated with diabetes may indirectly trigger kidney damage (Fig. 1).

## Historical overview of diabetic nephropathy classification and diabetic kidney disease

The initial classification of diabetic nephropathy stages, based on the progression of the disease in the individuals with type 1 diabetes, was introduced by Mogensen et al. in 1983 [3]. On this basis, a classification system relevant to type 2 diabetes was subsequently introduced in Japan in 1991. This classification was revised by the Joint Committee on Diabetic Nephropathy in 2001, leading to the current version published in 2014 [9, 17], with minor amendments added in 2023. The 2014 revision of classification [9, 17] incorporated considerations of the interplay between urinary albumin levels and eGFR on kidney outcomes, cardiovascular events, and overall prognosis from a cohort study in Japan [17]. This classification system was designed to be clinically comprehensive, including cases without albuminuria, thus enhancing its utility in clinical settings. However, this inclusivity has led to certain debates regarding whether all cases with kidney impairment can be definitively classified as having “diabetic nephropathy”. Some experts have also expressed discomfort with the direct transition from stage 1 (normal albuminuria) to stage 4 (the stage with severe kidney impairments) [9, 17].

## Introduction of diabetic kidney disease in the Japanese Clinical Guidelines 2018

The Clinical Practice Guidelines for Chronic Kidney Disease 2018 published by the Japanese Society of Nephrology [18] introduced the concept of diabetic kidney disease, which included diabetic nephropathy and other chronic kidney diseases (CKDs) in individuals with diabetes. However, its pathophysiology and diagnostic criteria remain undefined, characterizing all individuals with diabetes and kidney diseases as having diabetic kidney disease. However, the terminological similarity between “diabetic kidney disease” and “diabetic nephropathy” (especially in the Japanese language) has led to discrepancies among experts regarding the pathology, pathophysiology, and definitions of kidney impairment in diabetic kidney disease, as well as the pathological significance of the used of the word “diabetic” as an adjective.

## The incidence of reduced eGFR cases is increasing: could this be an undesirable effect of RAS inhibitors?

There is concern about why the number of cases with a reduced eGFR is increasing over time. The significance of RAS inhibitors in combatting diabetic nephropathy was established following reports on the efficacy of captopril for treating overt diabetic nephropathy in type 1 diabetes by Lewis et al. in 1993 [19] and on the utility of losartan for treating overt diabetic nephropathy in type 2 diabetes by Brenner et al. in 2001 [20]. Urinary albumin values are a crucial factor in determining the prognosis of diabetic nephropathy, and approximately 30 years after these reports, RAS inhibitors have been continuously prescribed with the aim of preventing kidney complications. However, long-term use of RAS inhibitors has also been suggested to potentially have adverse effects on the kidney. Abnormal proliferation of glomerular arteriolar smooth muscle cells and increased renin granule secretion in preclinical trials with long-term administration of angiotensin receptor blockers (ARBs) has been reported [21]. Furthermore, pathological analysis of the kidney arterioles in the normal parts of kidneys partially resected from patients with malignant tumors revealed that, while there were mild changes in patients with hypertension who were taking calcium channel blockers compared to normotensive patients, patients in the ARB-treated group exhibited narrowing of kidney arterioles, disorganized and marked proliferation of vascular smooth muscle cells, and enlargement of the vascular smooth muscle cells of the glomerular afferent arterioles [22]. These changes, potentially contributing to the suppression of glomerular hyperfiltration and the correction of glomerular hypertension, could even lead to glomerular ischemia. In addition, RAS inhibition has been associated with inducing acute kidney injury (AKI) [23], and AKI may lead to the AKI-CKD sequence in the individual with diabetes [24]. Regard with this, for individuals with diabetes without albuminuria, antihypertensive drugs other than RAS inhibitors can be now the first-line treatment as well. Approximately 20 years after ARBs began to be prescribed for most kidney disease individuals with diabetes, it is now necessary to consider their appropriate use. All clinical trials for individuals with kidney disease and diabetes have been primarily designed to use the maximum tolerable doses of RAS inhibitors. However, whether this baseline prescription of RAS inhibitors is suitable in the current model requires further discussion.

## Diabetic kidney disease in Japan: updating the corresponding Japanese term and concept

CKD is one of the most significant and important comorbidities in diabetes; however, worldwide, terminology regarding this important disease is chaotic. In KDIGO 2022 [25], the term “diabetic kidney disease” is not used to avoid the connotation that CKD is caused by traditional diabetes pathophysiology in all cases (however, they also state that the term “diabetic kidney disease” is entirely appropriate when this limitation is recognized). Additionally, “diabetic nephropathy” is not used because it is outdated and lacks a clear definition. Instead, all kidney complications in individuals with diabetes are referred to as “diabetes and CKD”.

However, it remains an open question whether treating CKD in individuals with diabetes is the same as treating CKD in individuals without diabetes for the following reasons.

- Diabetic nephropathy is undoubtedly a disease entity, as evidenced by its natural history [4, 5].
- Compared to individuals without diabetes, the individuals with diabetes generally exhibit a faster decline in kidney function [26, 27].
- Compared with those without diabetes, all kidney constituent cells (glomeruli, vessels, tubules, etc.) in the individuals with diabetes exhibit distinctive metabolic characteristics [28].
- It is believed that the pathology of the kidneys in the individuals with diabetes is influenced by a combination of related factors such as obesity, hypertension, metabolic abnormalities, and heart failure associated with diabetes [14, 15, 29].
- The impact of medications that have been used for many years in the individuals with diabetes cannot be disregarded [11–13, 22, 30].
- The effects of long-term survival, prolonged disease history, and aging in the individuals with diabetes are also significant factors that cannot be overlooked [11].

**Fig. 2:**
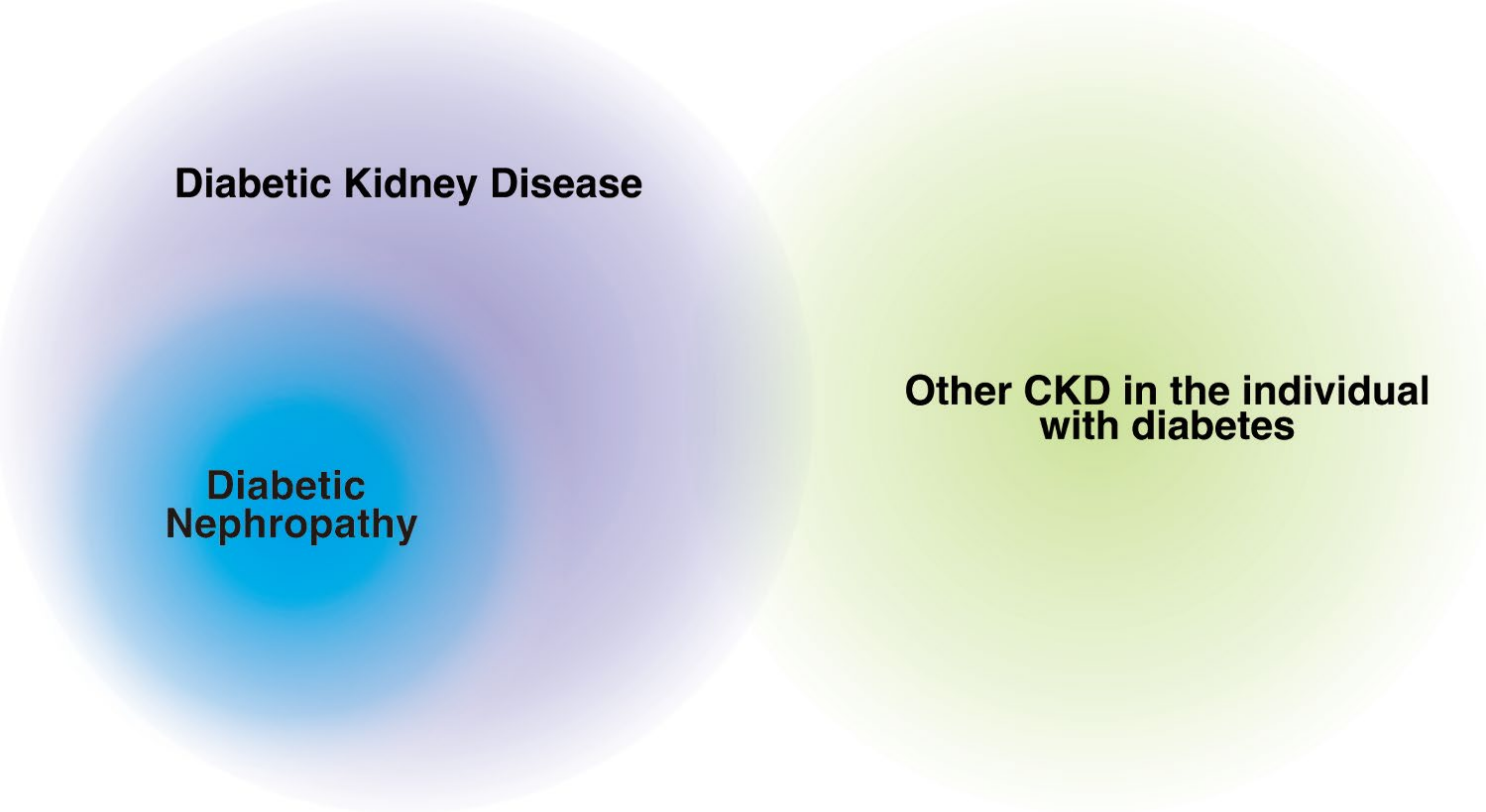
Relationship between diabetic kidney disease, diabetic nephropathy, and other CKDs in individuals with diabetes (modified from reference [31]) CKD in individuals with diabetes throughout their life, potentially influenced by diabetes and diabetes-relevant conditions, comorbidities, or treatments, is defined as diabetic kidney disease. Diabetic nephropathy, a key component of diabetic kidney disease, occurs specifically in diabetic cases. While other CKDs in individuals with diabetes are distinct from diabetic kidney disease. However, as the duration of diabetes extends, the impacts associated with the diabetic condition become increasingly pronounced, rendering the differentiation between diabetic kidney disease and other CKDs exceedingly challenging

Therefore, the Japanese Diabetes Society and the Japanese Society of Nephrology updated the corresponding Japanese term to describe diabetic kidney disease and clearly defined the concept of diabetic kidney disease [31] (Fig. 2). The major purpose of this update is to change the interpretation of the term “diabetic” in diabetic kidney disease from a “diabetes-essential condition” to a “diabetes-relevant condition” in the Japanese language.

Definition of Diabetic kidney disease: CKD in individuals with diabetes throughout their life, potentially influenced by diabetes and diabetes-relevant conditions, comorbidities, or treatments, is defined as diabetic kidney disease.

Inclusion criteria for diabetic kidney disease: the inclusion criteria for diabetic kidney disease followed the “CKD diagnostic criteria” for individuals with diabetes. Currently, it is an academic definition rather than a diagnosis, implying that diabetic kidney disease itself does not use diagnostic criteria. Here, given the increasing complexity of kidney damage in individuals with diabetes over time, “diabetic kidney disease” is defined, signifying collective efforts to elucidate each pathology within and find appropriate treatments accordingly.

Differentiation from other CKDs coexisting with diabetes: in individuals with diabetes, if there are other clear CKDs (glomerulonephritis, polycystic kidney disease, interstitial nephritis, etc.), they are not included in diabetic kidney disease (other CKD in the individual with diabetes). However, as diabetes duration increases, it is likely that the diabetic states contributes to the pathology of each case. The clear differentiation and contribution of diabetes becomes challenging with a longer disease history, which is often clinically observed. Even in such situations, it is necessary to differentiate, as much as possible, between diabetic kidney disease and other CKDs with existing specific interventions and cures (Fig. 2).

Kidney diseases interpreted as a diabetes-relevant condition: the Japanese term for the interpretation of diabetic kidney disease as a “diabetes-essential condition” has been changed to a “diabetes-relevant condition.”

**Fig. 3:**
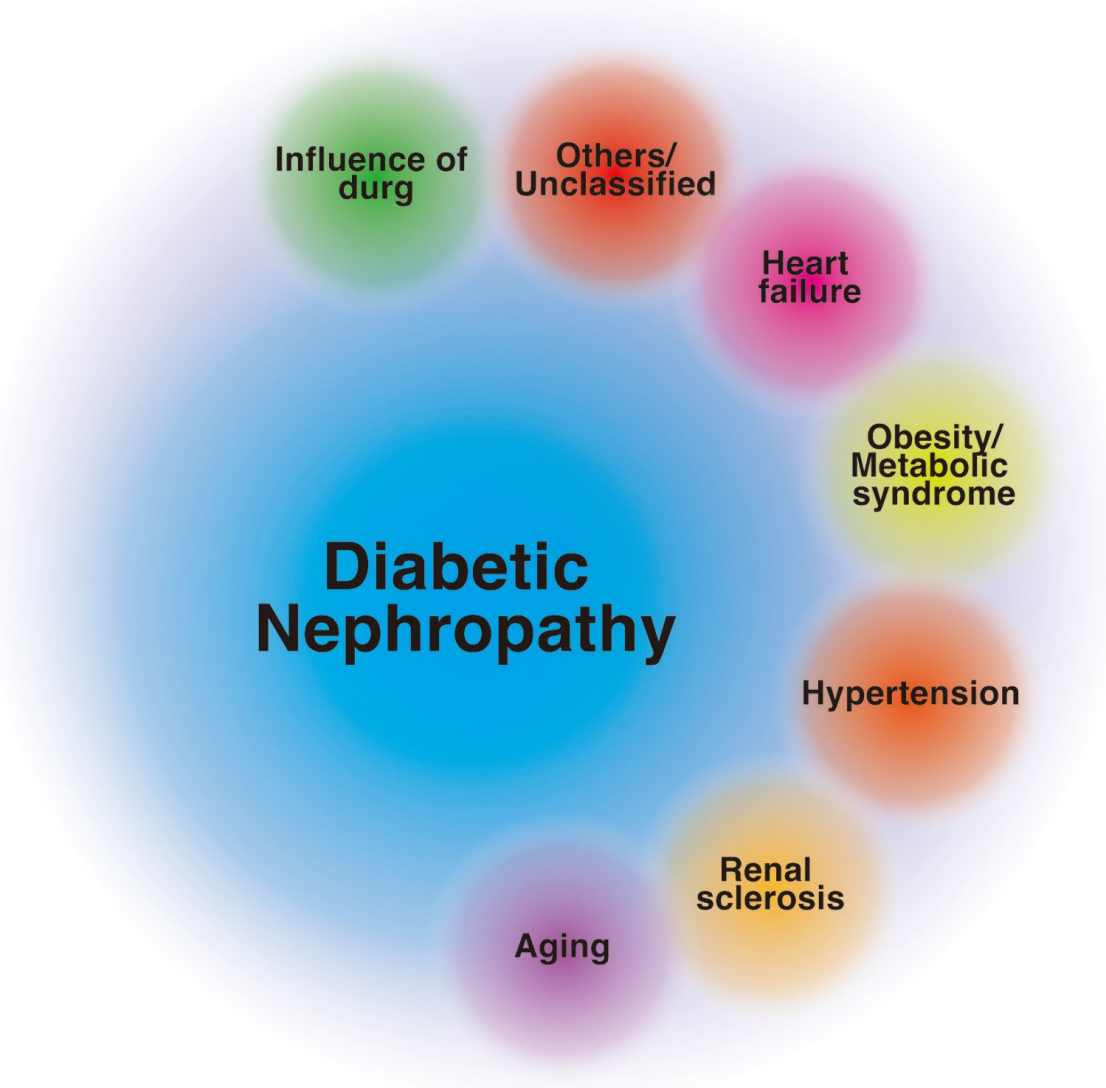
The possible structure of diabetic kidney disease (modified from reference [31]) Diabetic nephropathy, the form of kidney disease specifically associated with diabetes, is influenced by various diabetes-related conditions and treatments across an extended disease history. Currently, it is nearly impossible to precisely analyze the contribution of these factors. In clinical practice, the presence of urinary albumin, which is crucial for prognosis, serves as the primary indicator. Other conditions are also considered when determining appropriate interventions. Therefore, a deeper understanding of the pathophysiology of diabetic kidney disease is essential, along with the development of treatment strategies that distinctly differentiate between diabetic nephropathy and its various subtypes of clusters

Diabetic nephropathy and notable cases of diabetic kidney disease: from the natural history of diabetes, “diabetic nephropathy” is considered the most clinically significant diabetic kidney disease. In Japan, medically, the term diabetic nephropathy is utilized for a disease state that follows a typical course, as mentioned above. However, there is no solid definition beyond clinical staging, nor is there a perfect correlation between clinical stages and pathological images. Moreover, as the duration of diabetes increases, the contribution of comorbidities or relevant conditions to the kidney phenotype could be significant, yet the impact of these factors on the pathogenesis of the kidney phenotype remains unclear (Fig. 3). On the other hand, the current classification of diabetic nephropathy stages [9, 17] (with minor amendment in 2023) is already being utilized for patient education and guidance on the prevention of severe diabetic nephropathy, among other medical interventions and administrative guidance goals. The promotion of interventions focusing on urinary albumin is correct and has contributed to improving the prognosis of diabetic nephropathy. Confusion among individuals with diabetes, the stakeholders, is similar to putting the cart before the horse; therefore, patient education and guidance on the prevention of diabetic nephropathy based on the current stage classification should continue. Additionally, in the current Japanese classification of diabetic nephropathy stages, cases with a normal albuminuria phase and an eGFR of 30 ml/min/1.73 m^2^ to less than 60 ml/min/1.73 m^2^ are classified as stage 1. As indicated previously, the EMPA-Kidney trial [7] has shown that the decrease in the eGFR in cases with a normal albuminuria phase is minimal. However, in the normal albuminuria phase, “cases with an eGFR less than 45 ml/min/1.73 m^2^ [32], or those with an eGFR of 45 ml/min/1.73 m^2^ or more but less than 60 ml/min/1.73 m^2^ and a recent annual eGFR decline rate exceeding − 3 ml/min/1.73 m^2^/year [33]”should be considered at high-risk diabetic kidney disease and warrant monitoring.

## Conclusion

In this review, we outlined the concept and definition of diabetic kidney disease in Japan, as well as its background. Moving forward, further understanding of the pathophysiology of diabetic kidney disease is essential. It is crucial to distinguish between typical diabetic nephropathy and other conditions within diabetic kidney disease, leading to the development of diagnostic and treatment methods. Ultimately, these efforts must improve the prognosis of individuals with diabetes and kidney complication, who are the most important stakeholders; otherwise, such concepts and definitions would be meaningless. We are halfway through the journey of precision medicine for diabetic kidney disease, and we must promote the advancement of further clinical and basic research.
